# The Measurement and Uncertainty of a Calibration Standard for the Scanning Electron Microscope

**DOI:** 10.6028/jres.099.015

**Published:** 1994

**Authors:** J. Fu, M. C. Croarkin, T. V. Vorburger

**Affiliations:** National Institute of Standards and Technology, Gaithersburg, MD 20899-0001

**Keywords:** interferometer, precision, random error, systematic error, SRM, uncertainty

## Abstract

Standard Reference Material 484 is an artifact for calibrating the magnification scale of a Scanning Electron Microscope (SEM) within the range of 1000 × to 20000 ×. Seven issues, SRM-484, and SRM-484a to SRM-484f, have been certified between 1977 and 1992. This publication documents the instrumentation, measurement procedures and determination of uncertainty for SRM-484 and illustrates with data from issues 484e and 484f.

## 1. Introduction

**Fig. 1:**
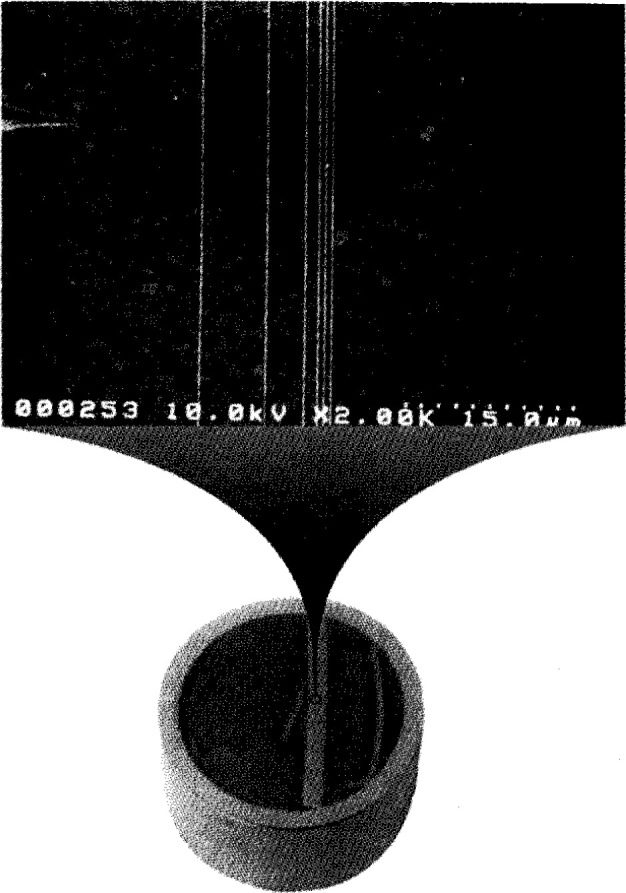
A micrograph of SRM-484f.

Standard Reference Material (SRM)-484 is a sample for calibrating the magnification scale of a Scanning Electron Microscope (SEM). The SRM consists of nickel layers separated by thin layers of gold. Individual samples are mounted so that the layers are viewed in cross-section and appear as thin gold lines in a nickel substrate. The distances or spacings between the gold lines are certified for calibrating the magnification scale of an SEM. The latest issue, SRM-484f, was certified for five spacings, nominally of 0.5 μm, 0.5 μm, 1 μm, 3 μm, and 5 μm. A micrograph is shown in Fig. 1. However, issues prior to 484f contained spacings between 1 μm and 50 μm.

**Fig. 2:**
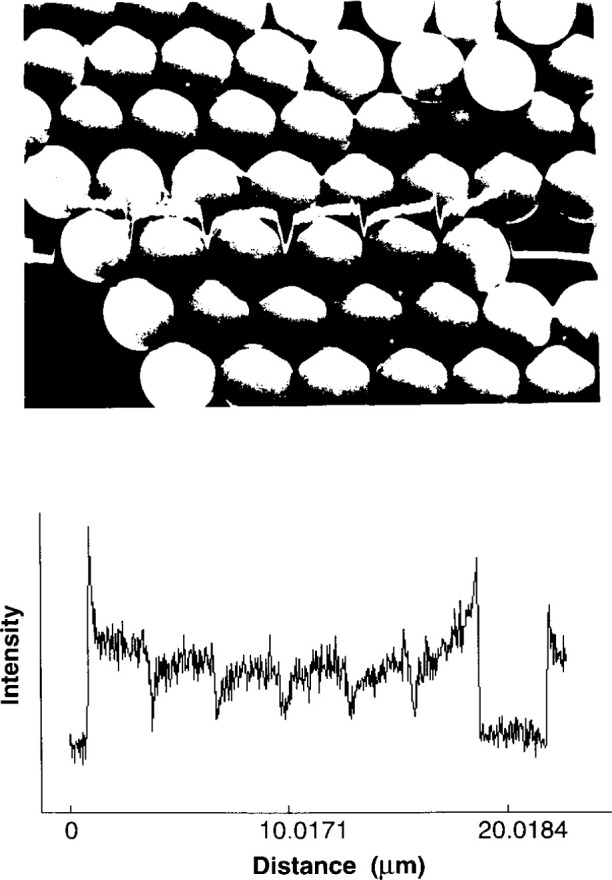
Micrograph of 3 μm latex spheres and the measured data of backscattered electron intensity versus distance.

SRM-484 was developed by Ballard at the National Bureau of Standards (now NIST) in 1977 [1]. The first three issues, SRM-484, SRM-484a, and SRM-484b, were calibrated using an electron microscope by comparing the micrograph of each sample against the micrograph of a master sample which had been measured with the NIST line-scale interferometer system [2]. In 1980, studies by Swyt, Jensen, and Hembree [3,4,5] led to the development of a system for direct calibration. This system, which combines a field emission scanning electron microscope with an interferometer system, has been used for calibrating SRM-484 since 1981 with only slight modifications over the years. It has also been used for confirming the diameters of polystyrene spheres with nominal values of 3 μm, 10 μm, and 30 μm. The approach is a pitch measuring technique as shown in Fig. 2. More in-depth detail can be found in Refs. [6], [7], and [8].

## 2. Physical Properties

**Fig. 3:**
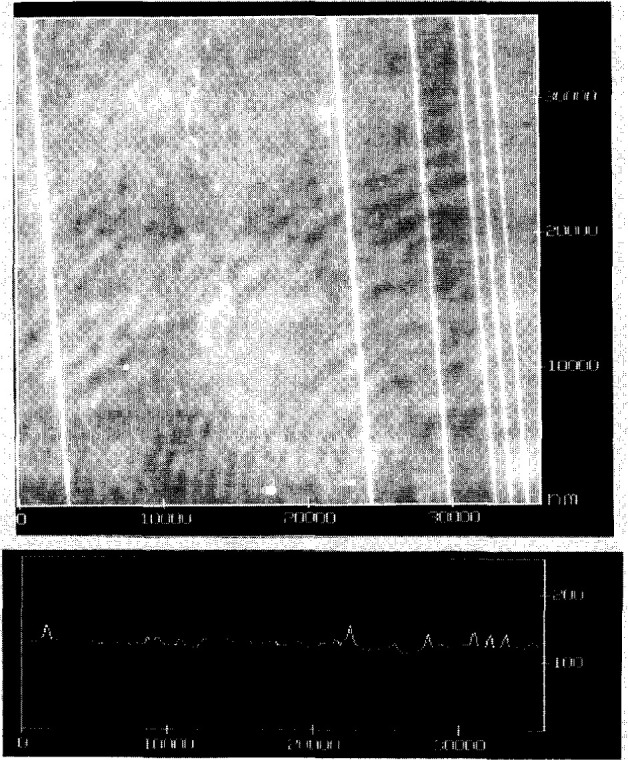
An AFM image of one of the SRM-484e samples. The section profile shows the height of the lines. The height of the left most line is ≈24.5 nm. The height of the third line from the left is ≈23.6 nm.

The fabrication process developed by John Young and Fielding Ogburn at NIST involves electroplating a layer of bright nickel onto the surface of a thin Monel sheet and subsequently electroplating alternate layers of gold and nickel to produce layers of desired thickness between the gold layers. The gold layer thicknesses are controlled to 200 nm or less. The sheet is vacuum heat treated at 265 °C for 16 h to relieve the residual stress in the layers. Several composite sheets have been produced by this process, each containing between five and ten spacings in the range of 0.5 μm to 50 μm. For each issue, individual samples of size 9 mm × 9 mm are sheared from a sheet and mounted sideways in a steel holder for metallographic polishing. Normally in a polishing process, soft material is removed faster than hard material, but images from a scanning tunneling microscope (STM), an atomic force microscope (AFM), and a stylus profilometer all reveal that, after polishing, the gold lines of the SRMs protrude about 30 nm above the nickel surface (Fig. 3). We speculate that a hard gold-nickel alloy may have been formed by the heat treatment, or the removal rate of nickel is faster than that of gold due to the chemo-mechanical effects in the polishing.

## 3. Instrumentation

**Fig. 4:**
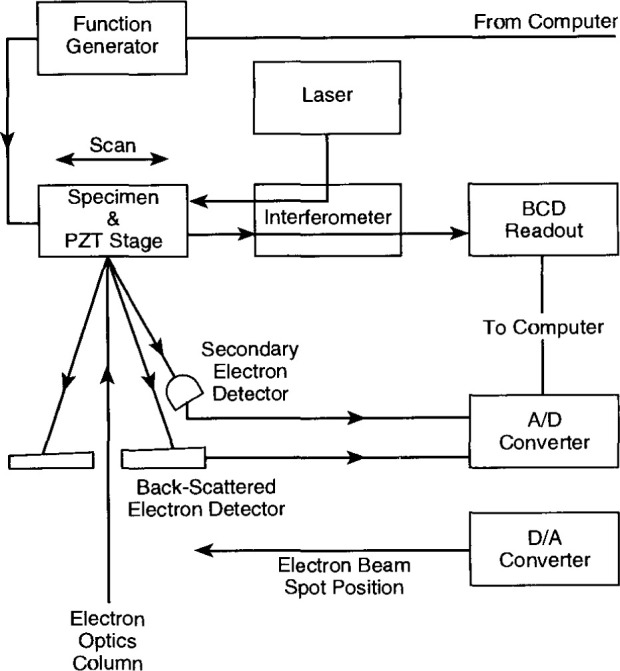
System diagram of the NIST metrology electron microscope.

The measuring system consists of a VG-HB50A field emission scanning electron microscope, HP5526A laser interferometer, piezoelectric displacement stage, wave generator and Digital MINC/DECLAB 23 computer^1^. A system diagram is shown in Fig. 4. A 30 kV, 5 × 10^−9^ A beam is used. Such a low beam current is necessary to minimize contamination marks on the SRMs. A scintillator back-scattered electron (BSE) detector, rather than a secondary electron detector, is used because of the strong material contrast between gold and nickel [9] for backscattered electrons. The vacuum in the sample chamber of the SEM is maintained at 3 × 10^−7^ Pa during calibration.

**Fig. 5:**
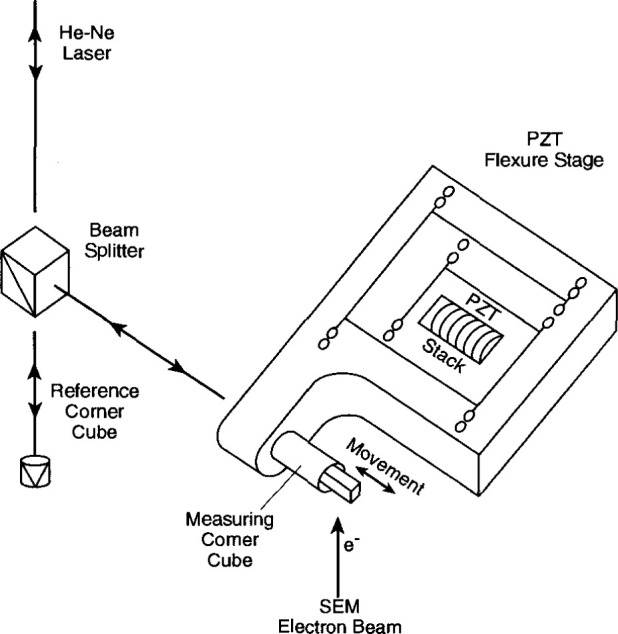
Schematic drawing of the optical system of the metrology electron microscope.

In ordinary SEM operation, the electron beam is scanned across the sample to obtain the image; however, for SRM-484, the electron beam is held stationary. The SRM is carried by the piezoelectric displacement stage [10] and scanned across the beam. The stage motion is driven by a high voltage power supply (not shown in Fig. 4) whose output is controlled by a programmable wave function generator. The reflector of the measuring leg of the interferometer is mounted on the stage. A polarizing beam splitter and the optics of the reference leg are held stationary on the platform adjacent to the stage. A schematic diagram of the optical system is shown in Fig. 5.

**Fig. 6:**
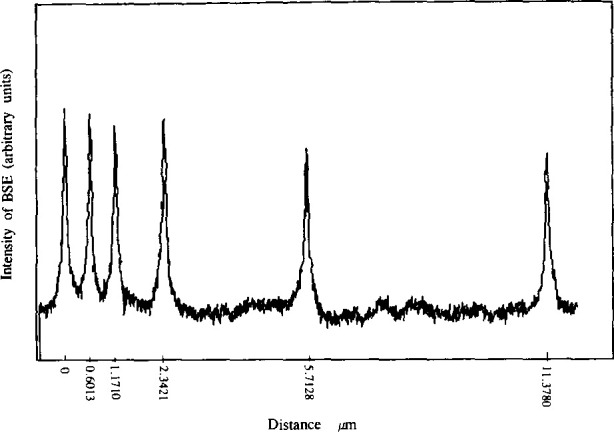
A plot of measured BSE signal versus distance for one SRM from issue 484f.

**Fig. 7:**
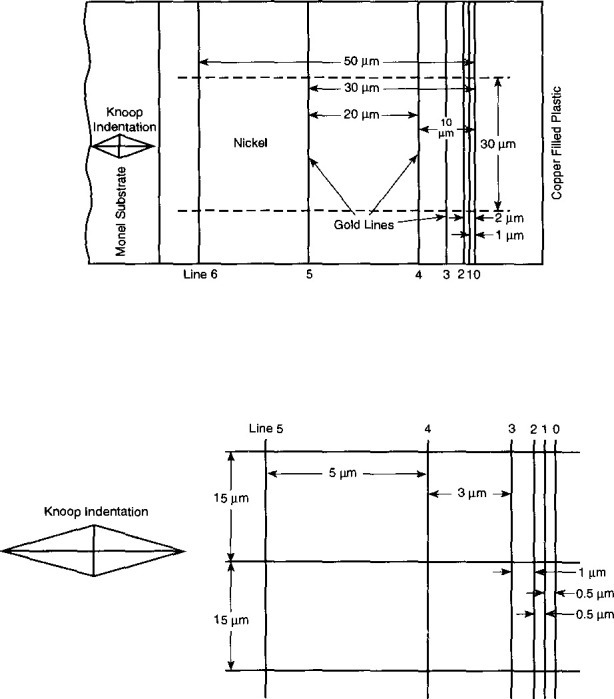
The lines pattern for SRM-484a-e (Top) and 484f (Bottom).

As the stage scans, the computer records both the displacement of the stage as measured by the interferometer, and the current of the backscattered electrons collected by the BSE detector. Each scan takes 48 s to complete during which time 2000 pairs of distance measurements and intensities are collected. The time interval between data points is approximately 25 ms. This is equivalent to a distance of approximately 6 nm for SRM-484f and 30 nm for the other issues which were measured at higher scanning speeds. The distance between the centers of any two gold lines is computed as the difference between position readings under the two corresponding intensity peaks. A typical plot of SRM-484f data is shown in Fig. 6. The peak-to-peak measurement technique produces an unbiased estimate of the spacing between the two gold lines. The measurement problem is quite different from the linewidth measurement problem [3,4,11] where an unbiased estimate of width requires a left-edge to right-edge determination. The pattern of lines for SRM-484 prior to 484f is shown at the top of Fig. 7, and SRM-484f is shown at the bottom. An example of certified spacings is shown in Table 1 for SRM 484f.

## 4. Measurement Procedure

**Fig. 8:**
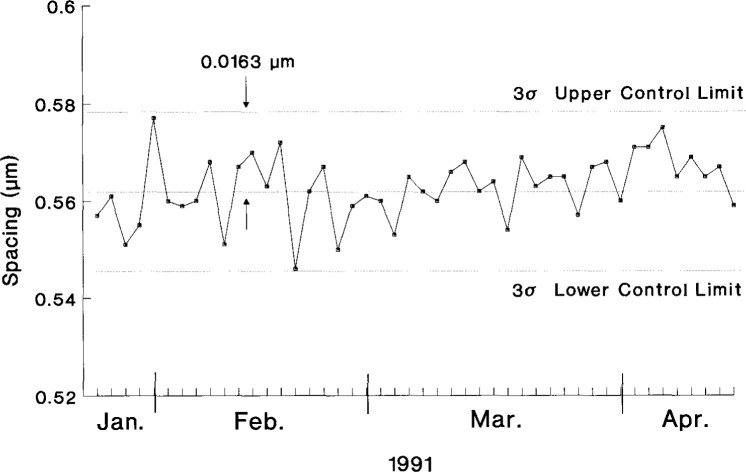
Control chart showing the 0.5 μm spacing measurements on the control sample for SRM-484f.

Although the measurement procedure has varied slightly from issue to issue, for the last four issues it has always involved multiple scans for estimating the effects of both instrumental error and non-parallelism of the gold lines. For issue 484e, each sample was scanned across the beam three times at each of three positions; for issue 484f, the number of positions was increased in order to check on parallelism, each sample being scanned across the beam nine times. The first three scans were at the same location along the Knoop indentation mark; the remaining six scans were at evenly spaced intervals that span 15 μm above and below the indentation mark. Approximately 150 samples were individually certified for each issue. During the period of calibration of the issue, which lasts approximately 3 months, a master sample, previously measured by the NIST line-scale interferometer system, and a control sample, selected at random from the samples in the current issue, are measured periodically in exactly the same manner as the SRM samples. The measurements on the control sample are made with sufficient regularity to cover the SRM certification in fairly even time increments and sample the range of experimental conditions. The history of measurements on spacing 0–1 of the control sample for SRM-484f is shown in Fig. 8.

## 5. Components of Uncertainty

The uncertainties quoted in the calibration certificate for SRM-484f and its predecessors were based upon an analysis of uncertainty arising from random and systematic components, as described by Eisenhart, Ku, and Collé [12]. Since the issuance of the calibration certificate, a new NIST policy and associated guideline document [13] were issued for the evaluation and expression of the uncertainties of NIST measurement results. This new policy, among other things, classifies uncertainty components according to whether they are evaluated by statistical methods or by other means, and deemphasizes the use of the terms “random” and “systematic.” Future calibration certificates and publications concerning the SRM-484 series will follow the new NIST guideline. However, the analysis here is based on the discussion of uncertainty in previous calibrations.

The total uncertainties for the measured line spacings are classified into random and systematic components. The random component of uncertainty depends on (1) instrumental precision, (2) the raggedness or lack of parallelism for the gold lines which causes disparities among positions on the sample, and (3) long-term measurement precision. The systematic component of uncertainty depends on the relationship between the measured value of line spacing and that realized through line-scale interferometry. A detailed description for each component is given below.

**Fig. 9:**
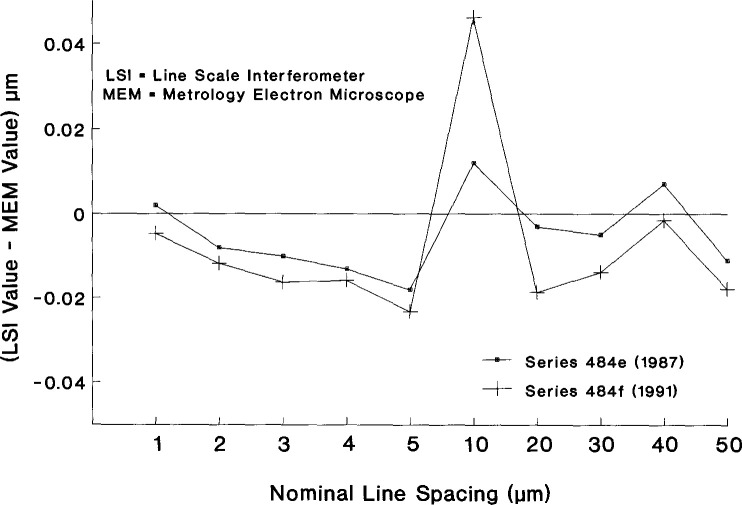
Difference between measurements made by line-scale interferometer and SEM on the master sample.

1. Instrumental precision is estimated from repetitive scans made at the same position on each sample. For SRM-484e, there are three scans at each of three positions and for SRM-484f, the only repetitive scans are at the center position on each sample. The measurement of the *i*th scan at the *k*th position is denoted by *x_ik_* (*i* = 1,⋯,*I*; *k* = 1,*⋯*,*K*). Estimates of instrumental precision are made according to
  $$s_{\text{inst}}={\left(\frac{1}{\left(I-1\right)}\sum_{i=1}^{I}{\left(x_{ik}-x_{\cdot k}\right)}^{2}\right)}^{1/2},$$
  where
  $$x_{\cdot k}=\frac{1}{I}\sum_{i=1}^{I}x_{ik}.$$
2. Between-position precision is estimated from the measurements at *K* distinct positions by
  $$s_{\text{position}}={\left(\frac{1}{\left(K-1\right)}\sum_{k=1}^{K}{\left(x._{k}-x..\right)}^{2}\right)}^{1/2},$$
  where
  $$x..=\frac{1}{K}\sum_{k=1}^{K}x._{k}.$$
  The estimates of instrumental precision are pooled [14] over positions and the *n* samples to obtain an overall estimate, ${\hat{\sigma }}_{\text{inst}}$, and the estimates of between-position precision are pooled similarly to obtain an overall estimate ${\hat{\sigma }}_{\text{position}}$ (the caret ^ symbolizes a statistical estimate from data). Any lack of parallelism is revealed in the standard deviation,
  $${\hat{\sigma }}_{\text{lack}}={\left({\hat{\sigma }}_{\text{position}}^{2}-\frac{1}{I_{0}}{\hat{\sigma }}_{\text{inst}}^{2}\right)}^{1/2}.$$
  For issue 484e, the value of *I*_0_ equals 3; for issue 484f, the value of *I*_0_ equals 1.2 to account for the unequal number of scans at the seven positions [15]. The results of the calculations for both SRM-484e and SRM-484f are shown in Table 2. A comparison of ${\hat{\sigma }}_{\text{inst}}$ and ${\hat{\sigma }}_{\text{position}}$ shows that ${\hat{\sigma }}_{\text{lack}}$ is significant for issue 484e, especially for the larger spacings, indicating a general lack of parallelism for the SRMs in this issue. For issue 484f, ${\hat{\sigma }}_{\text{lack}}$ is negligible indicating that, in general, parallelism is not a problem for this issue. However, an individual sample from issue 484f could have parallelism problems if *s*_position_ for that sample is excessively large; i.e., if
  $$s_{\text{position}}>\sqrt{F_{0.05}(6,2n)}\frac{{\hat{\sigma }}_{\text{inst}}}{1.2},$$
  where *F*_0.05_(6,2*n*) is the upper 5 percent point of the F distribution with 6 degrees of freedom in the numerator and 2*n* degrees of freedom in the denominator. Only 5 percent of issue 484f fell into this category as should happen by chance; nonetheless, samples with large standard deviations were examined and remeasured for confirmation.
3. A total standard deviation, *s*_t_, is estimated from the *M* calibrations on the control sample where the average value over all scans for the *m* th calibration is denoted by *y_m_* (*m*=1,⋯,*M*). For SRM-484f, the standard deviation is given by
  $$s_{t}={\left(\frac{1}{M-1}\sum_{m=1}^{M}{\left(y_{m}-y.\right)}^{2}\right)}^{1/2},$$
  where
  $$y.=\frac{1}{M}\sum_{m=1}^{M}y_{m}.$$
  The quantity *s*_t_, as shown in Table 2, accounts for both instrumental precision and long-term effects which cannot be controlled in the laboratory. For SRM-484e, the total standard deviation was estimated for each position and then pooled over positions so as not to include parallelism problems.
4. Systematic error relative to the defined unit of length was studied with measurements on a master magnification sample which has been measured several times with the SEM and several times with the NIST line-scale interferometer system. The line-scale system has an estimated (3*σ*) uncertainty of 0.01 μm. Differences between values obtained with the line-scale system and values obtained with the SEM help to identify systematic errors in either system. The uncertainty (3*σ*) of measured differences between the SEM and line-scale system is approximately 0.1 μm. The differences, as shown for issues 484e and 484f in Fig. 9, are within this uncertainty. For issue 484e, the differences are fairly randomly distributed about zero with a maximum of 0.012 μm and a minimum of –0.018 μm. For issue 484f, the differences are larger with a maximum of 0.046 μm for the 10 μm spacing.

The fact that the majority of the differences are in one direction for issue 484f may result from the treatment of the master sample. The SEM measurements are made on a smooth surface and the line-scale system requires an etched surface to increase contrast. The master sample was etched and measured with the line-scale system and then polished and measured with a SEM. The polishing process may remove enough material to slightly change the spacings relative to the etched surface. Although we do not treat the differences as being significant for the uncertainty statement, research in this area is continuing.

## 6. Certification and Uncertainty

Distances between the centers of gold lines are individually certified, and the certified region is located relative to a Knoop indentation. The certified value for each spacing is an average of all measurements, and the certification is valid within 15 μm of either side of an imaginary line extending from the Knoop indentation normal to the gold lines. The random component of uncertainty takes into account instrumental variability, long-term measurement fluctuation and any parallelism problem which affects the 15 μm region to either side of the center.

For issue 484e, the standard deviation of the certified value is

$$s={\left(s_{t}^{2}+\frac{1}{3}{\hat{\sigma }}_{\text{lack}}\right)}^{1/2}.$$

Because of the parallelism problems with the SRMs in this issue, the random component of uncertainty is reported as a statistical tolerance interval using an approximate Bonferroni [16] limit,

$$L=2s+\frac{v^{1/2}}{{\chi^{2}}_{0.05}}{\hat{\sigma }}_{\text{lack}}Z_{p/2}.$$

The first term in *L* represents a confidence limit and the second term represents an upper bound due to ${\hat{\sigma }}_{\text{lack}}$ where χ^2^_0.05_ is the lower 5 percent point of the *χ*^2^ distribution with *v* degrees of freedom, and *v*, the degrees of freedom associated with ${\hat{\sigma }}_{\text{lack}}$, is obtained from the Welch-Satterthwaite approximation [17]. The quantity *Z_p_*_/2_ is the upper *p*/2 percent point of the normal distribution with *p* = 0.05. The interpretation is that the interval defined by the certified value of spacing *±L* provides coverage for at least 95 percent of the spacings within the certified region at a confidence level greater than or equal to 95 percent.

For SRMs without parallelism problems (issue 484f), the random component of uncertainty is reported as 3 standard deviations, *L* = 3*s*_t_.

Historical uncertainties for all issues of SRM-484 are listed in Table 3. In all cases, the total uncertainty was reported as ± *U* where *U* is the linear sum of the random and systematic components of uncertainty or *U = L* + systematic uncertainty. The systematic uncertainty is considered negligible for issues 484e and 484f.

As an example, we also consider the uncertainty that would be quoted for SRM-484f under the new NIST guideline [13]. As discussed above, lack of parallelism and the uncertainties arising from other systematic effects have been considered as negligible. The combined standard uncertainty *u*_c_ is then purely statistical and given by *u*_c_*=s*_t_. The quoted uncertainty under the new NIST guideline is then the expanded uncertainty *U* = 2*u*_c_ = 2*s*_t_. Therefore, the expanded uncertainties *U* would be 2/3 of those given in Table 3 for SRM-484f.

## 7. Conclusion

It is essential for all SEM users to know the correct magnification of their instruments; the SRM-484 is a sample for calibrating the magnification scale of SEMs. The certified values of spacings between gold lines in a nickel matrix are measured with a metrology electron microscope and compared with line-scale interferometry. Properties of the SRM and the measurement system result in uncertainties of approximately 5% for 0.5 μm spacings and 1% for 50 μm spacings.

## Figures and Tables

**Table 1:** A typical set of certified values for SRM-484f

Spacing	Nominal value	Certified value
lines 1-0	0.5 μm	0.568 μm
lines 2-1	0.5 μm	0.579 μm
lines 3-2	1 μm	1.108 μm
lines 4-3	3 μm	3.308 μm
lines 5-4	5 μm	5.392 μm

**Table 2:** Component standard deviations, μm

SRM-484e						
Spacing	Instrument		Position		Total	
	${\hat{\sigma }}_{\text{inst}}$	df	${\hat{\sigma }}_{\text{position}}$	df	*s*_t_	df
1	0.029	1164	0.020	350	0.0167	117
2	0.031	1164	0.020	350	0.0193	117
5	0.031	1164	0.021	350	0.0199	117
10	0.031	1164	0.022	350	0.0210	117
30	0.032	1164	0.032	350	0.0239	117
50	0.032	1164	0.072	350	0.0361	117

SRM-484f						
Spacing	Instrument		Position		Total	
	${\hat{\sigma }}_{\text{inst}}$	df	${\hat{\sigma }}_{\text{position}}$	df	*s*_t_	df

0.5	0.012	318	0.012	954	0.0071	44
0.5	0.014	318	0.014	954	0.0067	44
1	0.013	318	0.014	954	0.0085	44
3	0.016	318	0.015	954	0.0117	44
5	0.018	318	0.017	954	0.0172	44

**Table 3:** Historical uncertainties for SRM-484

SRM issue	Nominal spacing (μm)	Uncertainty (μm)
SRM-484	1	±0.039
	2	±0.039
	3	±0.039
	5	±0.039
	50	±0.710
SRM-484a	1	±0.039
	2	±0.039
	3	±0.039
	5	±0.039
	50	±0.476
SRM-484b	1	±0.032
	2	±0.032
	3	±0.056
	5	±0.056
	50	±0.580
SRM-484c	1	±0.022
	2	±0.028
	3	±0.034
	5	±0.045
	10	±0.078
	20	±0.140
	30	±0.200
	50	±0.360
SRM-484d	1	±0.027
	2	±0.033
	3	±0.038
	5	±0.048
	10	±0.085
	20	±0.140
	30	±0.200
	50	±0.330
SRM-484e	1	±0.058
	2	±0.056
	5	±0.061
	10	±0.079
	30	±0.102
	50	±0.251
SRM-484f	0.5	±0.021
	0.5	±0.020
	1	±0.026
	3	±0.035
	5	±0.052

## Footnotes

Certain commercial equipment, instruments, or materials are identified in this papcr to specify adequately the experimental procedure. Such identification does not imply recommendation or endorsement by the National Institute of Standards and Technology, nor does it imply that the materials or equipment identified are necessarily the best available for the purpose.
